# Motivation and context of concurrent stimulant and opioid use among persons who use drugs in the rural United States: a multi-site qualitative inquiry

**DOI:** 10.1186/s12954-024-00986-z

**Published:** 2024-04-01

**Authors:** R. J. Fredericksen, R. Baker, A. Sibley, A. T. Estadt, D. Colston, L. S. Mixson, S. Walters, J. Bresett, X. A. Levander, G. Leichtling, T. Davy-Mendez, M. Powell, T. J. Stopka, M. Pho, J. Feinberg, J. Ezell, W. Zule, D. Seal, H. L. F. Cooper, B. M. Whitney, J. A. C. Delaney, H. M. Crane, J. I. Tsui

**Affiliations:** 1 University of Washington, Seattle, USA; 2 Oregon Health & Science University, Portland, USA; 3 University of North Carolina at Chapel Hill, Chapel Hill, USA; 4 The Ohio State University, Colombus, USA; 5 New York University, New York, USA; 6 Southern Illinois University School of Medicine, Springfield, USA; 7 Comagine Health, Portland, OR USA; 8 Tufts University School of Medicine, Department of Public Health and Community Medicine, Medford, USA; 9 University of Chicago, Chicago, USA; 10 West Virginia University, Morgantown, USA; 11 Cornell University, Ithaca, USA; 12 Tulane University, New Orleans, USA; 13 Emory University, Atlanta, USA

**Keywords:** Polydrug use, Concurrent stimulant and opioid use, Methamphetamine use, Rural

## Abstract

**Background:**

In recent years, stimulant use has increased among persons who use opioids in the rural U.S., leading to high rates of overdose and death. We sought to understand motivations and contexts for stimulant use among persons who use opioids in a large, geographically diverse sample of persons who use drugs (PWUD) in the rural settings.

**Methods:**

We conducted semi-structured individual interviews with PWUD at 8 U.S. sites spanning 10 states and 65 counties. Content areas included general substance use, injection drug use, changes in drug use, and harm reduction practices. We used an iterative open-coding process to comprehensively itemize and categorize content shared by participants related to concurrent use.

**Results:**

We interviewed 349 PWUD (64% male, mean age 36). Of those discussing current use of stimulants in the context of opioid use (n = 137, 39%), the stimulant most used was methamphetamine (78%) followed by cocaine/crack (26%). Motivations for co-use included: 1) change in drug markets and cost considerations; 2) recreational goals, e.g., seeking stronger effects after heightened opioid tolerance; 3) practical goals, such as a desire to balance or alleviate the effects of the other drug, including the use of stimulants to avoid/reverse opioid overdose, and/or control symptoms of opioid withdrawal; and 4) functional goals, such as being simultaneously energized and pain-free in order to remain productive for employment.

**Conclusion:**

In a rural U.S. cohort of PWUD, use of both stimulants and opioids was highly prevalent. Reasons for dual use found in the rural context compared to urban studies included changes in drug availability, functional/productivity goals, and the use of methamphetamine to offset opioid overdose. Education efforts and harm reduction services and treatment, such as access to naloxone, fentanyl test strips, and accessible drug treatment for combined opioid and stimulant use, are urgently needed in the rural U.S. to reduce overdose and other adverse outcomes.

## Background

The U.S. has entered the “fourth wave” of the opioid overdose epidemic, characterized by increased concurrent stimulant and opioid use beginning in approximately 2018 [1, 2]. This epidemic includes the use of both substances simultaneously and the concurrent use of one substance within hours of the other. In the U.S., among persons who use opioids, the use of stimulants, particularly methamphetamine, has increased dramatically [3, 4] with past-month use increasing from 9 to 44% among people who use heroin between 2015 and 2019 [4].

The consequences have been deadly. Opioids were involved in approximately half of the deaths in the U.S. in recent years among those who use methamphetamine [5]; of cocaine-involved deaths, nearly three-fourths involved opioids [6]. The high rates of overdose and death have been attributable to the adulteration of potent synthetic opioid analogs into methamphetamine, cocaine, and heroin, often taken unintentionally [7, 8], although recent studies have shown rising intentional use with an emerging preference for fentanyl over heroin [2, 9, 10], with stimulants used to offset the heavy sedation of fentanyl [11].

Concurrent use of stimulants and opioids may lead to worse health outcomes when compared to using either drug alone. Concurrent use of methamphetamine and heroin has been associated with more frequent injection and acute problems such as higher rate of nonfatal overdose [12], higher rates of blood clots, skin infections and abscesses, and endocarditis [13], and are more likely to have chronic conditions including hypertension, cardiac disease, chronic obstructive pulmonary disease, diabetes, kidney disease, hepatitis B or C, cancer, and HIV [14].

Until recently, the opioid overdose epidemic disproportionately impacted the rural U.S. population [15, 16]. Methamphetamine use rates are higher in rural compared to urban areas [14, 17], and rurality has been identified as a risk factor for use of heroin and methamphetamine together [4]. Among people who use drugs (PWUD), a 63% prevalence of using both methamphetamine and opioids has been observed in rural areas [12]. In general, factors for increased vulnerability to drug use among rural populations include low employment, poverty, less access to mental health services and to substance use treatment, and lower educational attainment [18]. Qualitative studies of motivations for dual stimulant and opioid use in rural populations illustrate the role of economic and associated mental health factors [19, 20] as well as other factors including intensive physical demands found within rural labor markets [20] and the need to remain productive [21].

To date, most qualitative studies of dual stimulant and opioid use have been conducted in urban populations [22], and most studies in rural populations have been small in sample size and restricted to specific regions [19, 20, 23]. Using interview data from 8 geographically diverse sites that comprise the National Institute on Drug Abuse (NIDA) Rural Opioid Initiative (ROI), and the benefit of a large sample size, we sought a fuller understanding of motivations and contexts for opioid and stimulant co-use use across the rural U.S.

## Methods

### Background

The ROI is a consortium of research studies funded in 2017 by NIDA to better understand the health impacts associated with the opioid crisis in rural parts of the United States (http://ruralopioidinitiative.org/) [24]. ROI consists of 8 sites spanning 65 counties across 10 states: Illinois (IL), Kentucky (KY), northern New England (NE, including Massachusetts, New Hampshire, and Vermont), North Carolina (NC), Ohio (OH), Oregon (OR), Wisconsin (WI), and West Virginia (WV). The initiative included standardized qualitative interviews across all sites with PWUD to better understand life history of opioid and other drug use, its context, and circumstances influencing health behaviors. Sites sent interview data to the Qualitative Core of the University of Washington Data Coordinating Center (UW-DCC) for data management and to centralize coding efforts.

### Interview guide development

We developed a standardized semi-structured interview guide in collaboration with the ROI Qualitative Methods Workgroup, which consists of researchers with qualitative method expertise across ROI sites. Content areas included but were not limited to current and past unregulated opioid and other drug use, experiences with overdose, local changes in drug use patterns, harm reduction, and access to substance use treatment. Questions eliciting experiences with stimulant use included: first use of any drug; first injection drug use (IDU); current IDU, change in use of current injected drug over time; and differences in IDU preparation behavior between drug types. We note that the focus of the interview was on initial and current opioid and injection drug use, as interview guide development preceded the widespread use of stimulants in rural areas. Stimulant use was asked primarily in the context of current IDU.

Each site received approval from a local institutional review board and participant privacy was protected by a federal Certificate of Confidentiality.

### Participant recruitment and data collection

We recruited PWUD to participate in a 60 to 90 min interview from 2018 to 2019. All participants had used non-prescribed opioids and were at least 18 years of age. Participant eligibility varied slightly between sites due to regional differences in drug use (Table 1). Participants were recruited from community-based programs, and at some sites, street outreach. Interested persons were administered informed consent per local site IRB protocols. Seasoned qualitative researchers conducted interviews in-person at each site, which were digitally recorded and transcribed verbatim. Depending on the site, participants were compensated $25-$50 for completing the interview.

**Table 1:** Criteria for questionnaire and qualitative interviews

Site	Questionnaire Criteria	Qualitative Interview Criteria	Qualitative interview dependent on completion of questionnaire?	Qualitative recruitment method
IL	Age ≥ 15 English speaking Injected drug to get high in last 30 days OR used any opioid by any route in last 30 days Not currently intoxicated Passes drug screening test	Accepts referral to Harm Reduction services	Yes	Questionnaire
KY	Age ≥ 18 English speaking Current resident in 12 county area Injected drugs or used opioids to get high within past 30 days Urine drug screen to verify drug use	Same as questionnaire	Yes	Questionnaire
NC	Age ≥ 18 English speaking Current resident in study area and intends to stay 12 months Opioid injection or methamphetamine injection in past 30 days Verification by stigmata or appropriate description of injection practices	Indication in questionnaire of having injected painkillers or heroin	Yes	Questionnaire
NE	Age ≥ 18 English speaking Injected drugs or used opioids to get high within past 30 days (self-report) Living within 11 rural counties (MA, NH, VT) Can provide informed consent	People who inject drugs and/or use opioids from 11 rural counties	No	Convenience sample of in-depth interview participants through street outreach, venue-based recruitment, and respondent driven sampling from larger study
OH	Age ≥ 18 Current resident of 3-county study area Have used heroin or prescription opioids OR have injected any type of drug to get high in the past 30 days Can provide informed consent	50% male/female 50% accessed health-or drug-related services in the past year Subsamples of individuals who have recently transitioned to injection and women with experiences with NAS	No	Community partners and key informants
OR	Age ≥ 18 English speaking Live in the study area Have injected drugs or report recreational opioid use without injection in the last 30 days Willing to provide consent for risk survey and future linkage of biologic and survey data to administrative data	Any PWUD living in study area	No	Advertisement in community-based and service locations; direct recruitment by service provider and outreach staff
WI	Age ≥ 15 English speaking Injected any opioid drug in the past 1 month Reside in a rural community	Same as questionnaire	Yes	Questionnaire
WV	Age ≥ 18 English speaking Current resident of one of 7 counties of study area Injection in past 30 days	Same as questionnaire	No	Direct recruitment by outreach staff and service providers of dynamic individuals well-known in the IDU community as "seeds" to recruit other PWID

### Analysis

We conducted preliminary coding to categorize data by interview topic areas and lines of inquiry using the Dedoose qualitative software program (v. 09.0.62). Upon retrieval of the data related to stimulant use, we developed a thematic coding scheme based on the principles of Grounded Theory analysis [25, 26] in which two investigators each independently used an “open-coding” process to comprehensively itemize and categorize content stated by participants related to stimulant use. The two open-coders reconciled differences in categorization, facilitated by the UW-DCC Qualitative Core leadership, and established a set of fixed codes for application to interview content. Another set of coders independently applied the fixed codes, again meeting to reconcile differences in interpretation to ensure consistency in application. Finally, we conducted a qualitative memo-ing process with two independent coders to summarize content within each fixed category and met to establish interpretive agreement (Table 2).

**Table 2:** Demographic characteristics of interview participants

		**Total**	**Ilinois**	**Kentucky**	**North Carolina**	**New England**	**Ohio**	**Oregon**	**Wisconsin**	**West Virginia**
**Participants**		**349 (100%)**	**22 (6%)**	**57 (16%)**	**65 (19%)**	**22 (6%)**	**26 (7%)**	**52 (15%)**	**60 (17%)**	**45 (13%)**
Male		194 (64%)	14 (64%)	35 (61%)	34 (52%)	10 (45%)	15 (58%)	28 (54%)	33 (55%)	25 (56%)
Average age		36	37	35	36	33	37	39	35	38
Race										
	White	213 (61%)	20 (91%)	56 (98%)	15 (23%)	15 (68%)	–	49 (94%)	58 (97%)	–
	Black	2 (1%)	2 (9%)	0 (0%)	0 (0%)	0 (0%)	–	0 (0%)	0 (0%)	–
	Native American	8 (2%)	0 (0%)	0 (0%)	5 (8%)	1 (5%)	–	1 (2%)	1 (2%)	–
	Mixed race	5 (1%)	0 (0%)	0 (0%)	2 (3%)	0 (0%)	–	2 (4%)	1 (2%)	–
	Other	1 (0%)	0 (0%)	1 (2%)	0 (0%)	0 (0%)	–	0 (0%)	0 (0%)	–
	Not given/ asked	120 (34%)	0 (0%)	0 (0%)	43 (66%)	6 (27%)	–	0 (0%)	0 (0%)	45 (100%)

## Results

We coded 1,672 excerpts from 349 PWUD interviews (64% male, mean age: 36 years).

Of PWUD who discussed current use of both stimulants and opioids (n = 137, 39%), the stimulant most mentioned was methamphetamine (78%) followed by cocaine/crack (26%).

Among those who reported recent use of both stimulants and opioids, some reported using them simultaneously, while others reported using the sequentially within a narrow time frame. Simultaneous use of both stimulants and opioids was discussed by 65 participants (47% of n = 137), and 73 participants (53% of n = 137) reported non-simultaneous use of the substances within a narrow time frame (e.g., taking a stimulant hours later to moderate the effect of an opioid, or vice versa). The dual use of methamphetamine and heroin was the most mentioned combination of drugs for simultaneous stimulant and opioid use (54% of 137) followed distantly by methamphetamine and pain pills, and cocaine and heroin (each 11% of 137). For non-simultaneous use in a narrow time frame, methamphetamine and heroin was again the most-mentioned combination (27% of 137), followed by methamphetamine and buprenorphine/naloxone (15%), methamphetamine and pain medication (14%), and methamphetamine and multiple opioid types (12%).

Motivations for stimulant use among persons who use opioids included: 1) change in drug markets and cost considerations, 2) recreational goals, such as using one drug to enhance effects of the other, to create a unique effect, or to achieve a heightened psychoactive effect after increased tolerance to opioids, 3) practical goals, such as balancing drug effects or alleviating each drug’s unwanted effects, including the use of methamphetamine for opioid overdose reversal or for managing opioid withdrawal symptoms; 4) functional goals, such as maintaining ability to work.

### Changes in drug markets and cost considerations

While a confluence of several factors increased stimulant use among participants, in every region, this was underscored by a dramatic increase in the availability of methamphetamine:

…meth is everywhere. And it’s like I can’t even walk down the street without someone stopping and asking if I can find “fast” [methamphetamine] or whatever. (Man, 32, Wisconsin)

…the opioid problem was big. But now methamphetamines…inside [my county] ismassively taking over. (Man, 34, West Virginia).

Participants commonly described limited access to prescription opioids as a driver to other drugs:

Since they’ve made it harder to get prescription [opioid] medication, people have turned to heroin...or crystal meth. (Man, 36, West Virginia)

…you saw prescription pills not being available anywhere near as much and that’s when heroin started to really boom and then 3, 4 years ago is when you saw the huge boom from crystal meth (Man, 39, Wisconsin)

Opioids and cocaine were often described as cost-prohibitive, while methamphetamine was relatively cheap and abundant. For cost reasons, participants typically began substituting methamphetamine in place of pills or heroin:

I would buy [methamphetamine], because I mean, it was so much cheaper than $200 a day on [opioid] pills. $200 would last a month [for methamphetamine], you know what I mean? (Woman, 33, North Carolina)

A gram of heroin costs about anywhere from $160 to $200 and a gram of meth is about 50 bucks…much cheaper, that’s why a lot of people choose it over cocaine too. I’ll try to use [heroin] sparingly…but I’ll smoke meth all day throughout the day. (Man, 40, Illinois)

Regardless of region, diminished availability of prescription opioids, and an increase in availability of methamphetamine (and in many areas, heroin) set the stage for dramatic changes in type and frequency of drug use at the community level, as summarized by one participant:

People went from drinking, smoking weed…eating Percocet, doing a line of coke here and there to doing heroin to replacing the Percocet with heroin and then replacing the cocaine with methamphetamine. (Man, 24, New England)

### Recreational goals: Seeking combined drug effects and stronger drug effects after heightened opioid tolerance

Participants described pursuit of a combination of psychoactive and physiological effects that were not attainable from either stimulants or opioids alone. Some described this in terms of a synergistic effect; others, in terms of a progression of desirable sensations. Many participants described using both drugs together to produce a unique effect, a euphoria that combined the best of each drug:

When you do them together [heroin/meth], it’s just to get a certain high. It’s a certain kind of rush. (Man, 25, Oregon)

It’s like [methamphetamine] goes up and [heroin] goes down, and you have to try to not necessarily meet in the middle, but get a good high somewhere in there. (Man, 29, Oregon)

Participants described simultaneous use of stimulants and opioids in terms of a desirable progression of sensations:

…when you do [meth and heroin] both together, your heart starts racing. Then you feel the warm feeling like your pain going away. (Man, 31, Kentucky)

I mix…a little bit of meth and heroin and coke. You get everything, and usually after you shoot up…then you get this fucking rush, and then all of a sudden you’re just buzzing from the heroin for hours…then, when you finally get out of that feeling, you’re fucking high as a kite on meth and coke. (Woman, 33, Oregon)

For those that did not inject the drugs simultaneously, there was a desire to control the timing of sensations. The most common preference was to start with opioids and follow with a stimulant:

I’ll do one and then do the other one. I’ll do the heroin first so I can get that good feeling. And then I’ll do the [methamphetamine] so I can take off. If I do meth first, then I have a hard time feeling the heroin. (Man, 38, North Carolina)

I like the rush from the heroin first. [Doing meth first] usually starts like you’re all warm, and then [makes fake retching noise]. It’s gross. (Man, 63, Oregon)

Finally, a common refrain among participants was a determination to pursue the combined effect even despite intense discomfort. In the words of one participant:

I’ll never forget the first time I [mixed fentanyl and meth], ... I had a script of fentanyl patches and I mixed 100 microgram patch, what I drew out of that, with a half a gram of meth, and I’ll never forget. I did that shot and I had to put a hand on each side of the wall because I literally felt like my brain was ripping in half and didn’t know which way I wanted to go. But whenever it came back to be tolerable, I just was whew, and it was like okay. I can do this….it’s an overwhelming euphoric effect. It’s almost like overload, literally. (Woman, 43, North Carolina)

Participants described building tolerance to their opioid of choice and mixing stimulants and opioids to re-experience the psychoactive effects felt when they initiated single drug use:

I couldn’t get high [on heroin] without the coke cause I had such a high tolerance. Even if I did 10 bags, I just didn’t get high anymore. So…you get high from the coke. (Man, 29, New England)

Upon reaching tolerance to opioid pills, one participant echoed others in describing the pursuit of a high by any means necessary, combining substances to simply get as intoxicated as possible:

After I had got my fill of the pain pills, I had started going to heroin. That was my favorite drug out of anything. Heroin spiked with cocaine; heroin was my thing. I had shot up in my neck. After I had moved back, I was still going down that same path. I was still shooting up. If it wasn’t heroin, it was meth. If it wasn’t meth, it was ice [smokable methamphetamine]. If it wasn’t ice, it was whatever I could get my hand on that was injectable. That’s what I used and my tolerance, like I just ... There were times that I wasn’t high and I wanted to get higher, so I would just do all these chem-, like all these substances together to get as high as I could. (Woman, 26, West Virginia)

### Practical goals: balancing drug effects or alleviating unwanted drug effects

Participants referred to the use of stimulants and opioids in terms of achieving or restoring some kind of ‘balance’, for example, offsetting the unwanted effects of either the stimulant, the opioid or both. The most profound example of attempting to balance effects was the common strategy of using methamphetamine to reverse opioid overdose:

We’d been out of pills and heroin for a few days and I was really sick. A friend brought a friend, brought over a bunch of Klonopin...I was so sick that I made up a bunch of Klonopins and Dilaudids and shot them together, and somehow it just put me out for three days. They came to see me the second day, and my lips were turning blue and I just was barely breathing. So they went out and they got some meth and go, “Here, take this.” “Okay, yeah.” That buzzed me pretty good. (Man, 30, Oregon)

We’d mix a lot, like do heroin and put meth in it….it’s considered “speedballing,” but some people do it ‘cause it’s safer…they won’t fall asleep, because they’ve got the meth to counteract the fentanyl a little bit. That’s what a lot of us think…I mean, I honestly don’t know if it works that way, but it seems like it does, ‘cause [boyfriend] has always done it that way with mixing it, and then one day he didn’t mix it…he didn’t OD, but he almost did. (Woman, 36, North Carolina)

Participants described using stimulants and opioids simultaneously or in succession (e.g., within a few hours to a few days) in an attempt to alleviate unwanted side effects of the other drug. Typically, stimulants were used to “wake up” from using opioids:

… pain pills and cocaine, the mixture of the two, give off a different high. Because pain pills…give you what they call a nod. It’s like a down, a sleepy sensation. And the coke was always an up, upper like and you could do the two together and you’ll just stay up for longer periods of time without falling asleep. (Man, 45, West Virginia)

…if I did too much heroin and I’m tired, I’ll do some meth and it wakes me up. (Woman, 26, Oregon)

Stimulants were also used to offset feeling of nausea from opioids:

I’ve injected Opanas [opioid medication]. But just a little bit of them, and it was always with some speed, because they make me sick. Even taking Lortabs makes me sick, queasy, want to puke. (Man, 37, North Carolina)

At the same time, opioids were used to mitigate the effects of stimulants:

The heroin would make me relax from the crack. (Man, 38, Illinois)

Only reason why I do heroin is because I need to go to sleep because of [methamphetamine]. (Man, 21, North Carolina)

Finally, participants typically described using stimulants, particularly methamphetamine, to cope with acute withdrawal symptoms from opioids. In the words of two participants:

There’s been times before where I’ve been really bad off on the pills and couldn’t get any, so I’d do meth and it would make me not withdraw and I’d be super, duper high off the meth for three days. Then, three days later I’d finally sleep and then my withdrawals would be gone pretty much. Totally withdrawn. I wouldn’t need the pills as much. (Man, 25, North Carolina)

I usually would do [methamphetamine] when I start feeling fatigue or really sick or whatever from not having heroin (Man, 28, Oregon)

### Functional goals

Participants were motivated by functional goals to use both stimulants and opioids. This included a goal of improving day-to-day functioning, often in the context of employment. Some sought a mix of feeling both energized and pain-free:

When you mix [methamphetamine and heroin]…it’s like you have energy, but you feel good. It’s like you don’t hurt, but you have energy. If you do just methamphetamine, there’s a lot of creaks—it’s like an old house—like your body just hurts. If you throw heroin in, then it’s like you get the energy without having to be the old house, without having to be like, “Oh, every bit of me hurts.” (Woman, 30, Oregon)

[Using meth and heroin] absolutely works, it kills pain…it’s going to elevate your mood, take away your depression…all the stuff that stimulants do. (Man, 48, North Carolina)

Participants noted the use of methamphetamine allowed them to continue to function and be productive:

Heroin is my drug of choice…I do speed to fucking function. (Man, 55, Oregon)

…it started off being that my drug of choice would be the opiate, but then I would do just a little bit of meth just to keep up with whatever I had to do that day. Like chores and like I had to help out with my dad. I had to do this or that and so just a little bit of meth would be helpful…as long as I get it in me I’m fine. (Woman, 49, North Carolina)

Many participants who used opioids noted the role of using methamphetamine to function for their employment, which typically involved physical labor:

I was running this landscape crew in Tennessee and would start every day by taking half a gram and dropping it in my Rockstar and drinking that…it keeps you awake. (Man, 25, North Carolina)

I do janitorial work…if I have to go to work, I’m going to do meth to get up. I’m going to work and get my job done, and then if I want to come down, I’m going to do heroin. (Man, 38, Oregon)

I do carpentry and odd jobs…anything from mowing yards to roofing to building houses…whatever. I try to find something to make money every day. You’ve got to live. It’s pretty physical work. But I can do one shot of meth, and I can be up for three days and nights constantly doing work, work, work. (Man, 46, Kentucky)

For many, financial hardship played a role in the use of methamphetamine. In the words of one participant:

I had three weeks to get in my power [bill] and everything before the snow was hitting the ground…my mom, my wife and everybody would have been without power, without heat. So I worked for about three weeks straight, 16 to 20 hour days, and meth was about the only thing that kept me able to do it. (Man, 33, Oregon)

One participant who used heroin and methamphetamine daily felt he needed methamphetamine to meet the physical demands of his employment, and spoke of limited opportunities for change:

I’ll do meth in the morning and then I’ll do heroin at night…I use meth pretty much at least five days a week. I use it, basically…as a tool to help me. I work a lot, and my job is demanding for hard work….I’m not 25 years old anymore. I work in a mill. My job is super fast-paced. Because of my record and stuff, I just can’t go and get the type of job that you have, or this or that. I have eight felonies on my record. I just can’t go and become anything I want to become anymore. (Man, 35, Oregon)

## Discussion

In this large multi-site sample of PWUD living in the rural U.S., discussion of stimulant use among persons who used opioids was highly prevalent, corroborating what has commonly been named the “fourth wave” of the opioid overdose epidemic. Reasons for dual use were diverse. Changes in local drug access set the stage, primarily higher local availability of methamphetamine, which was lower in cost than opioids, coupled with the persistence of available heroin. Many were motivated by the unique euphoric psychoactive effects attainable from combining both substances, and the ability to tailor the progression of sensations. Several participants described having acquired an increased tolerance to opioids over time that prevented the desired euphoric feelings they had experienced in the past and sought stimulants for simultaneous use with opioids to augment or bolster these feelings. Participants also sought practical goals, such as to mitigate symptoms of opioid withdrawal, but most commonly to offset the unpleasant effects of either drug. This often created an ongoing cycle of attempts to self-medicate alternating drug-related side effects. The combination was at times used to help function in daily life, particularly in employment. Notably, methamphetamine was regarded as a tool to avert or reverse opioid overdose.

Our qualitative study is the largest and most regionally diverse sample of people who concurrently use stimulants and opioids in the rural U.S., corroborating findings in smaller and/or non-rural studies that have identified drivers of concurrent use, such as the desire for unique psychoactive effects [20, 21, 27], to offset or balance the adverse physical and psychological effects of the other drug [3, 4, 20, 28], and to alleviate opioid withdrawal symptoms [20, 27, 29]. We note that three of our findings appear particularly salient to the rural U.S. context, and less prevalent in studies limited to urban populations. First, our participants described significant changes in local drug availability as a key driver for their methamphetamine use. Methamphetamine was less expensive and described as more readily available than opioids, aligning with findings from smaller rural U.S. studies [19, 21] and a large national sample [3]. Second, in contrast to studies limited to urban populations, participants often described functional goals, such as “having energy and being pain free”, in order to be productive in daily activities including employment, echoing findings elsewhere [21, 23]. Such goals may be particularly salient in the context of rural life, in which jobs are more likely to involve physical labor [30] rendering this combination of effects critical to livelihood. Third, for several of our participants, stimulants were thought of as a tool for reversing or pre-empting opioid overdose, a phenomenon consistent with findings in smaller qualitative studies [21, 31] but not described in known studies of PWUD in non-rural U.S. areas.

We note the importance of the use of stimulants as a practical tool for our participants. Many participants utilized methamphetamine to avoid opioid overdose and death, but also to soften or bypass the discomfort and cravings that accompany opioid withdrawal. For many, stimulant use offered the hope of being both pain-free and functional; for others it was described in terms of attempting positive engagement in their daily lives through increased energy and productivity. At the root of these motivations are clear instincts for survival: self-preservation, a desire to transcend circumstances, the pursuit of a better quality of life. These instincts may be leveraged as opportunities to engage PWUD in utilizing and publicizing evidence-based methods of harm reduction, as well as an entry point for substance use treatment.

The rural context, however, poses many barriers to such opportunities. There is limited access to relevant harm reduction measures such as test strips for screening substances for fentanyl adulteration prior to use, naloxone for opioid overdose reversal, and substance use treatment, including behavioral health services and treatment with medication for opioid use disorder (MOUD) [32–36]. Treatment access in rural areas, already challenging due to long distances and limited options [32, 37–40] is further complicated by the complex issues surrounding treatment of concurrent stimulant and opioid use. Factors contributing to this void include lack of a current pharmacological intervention for treating stimulant use disorders; unwillingness of many buprenorphine prescribers to treat persons who also use methamphetamine (Korthuis 2021); and the negative impact of stimulant use on retention in treatment with MOUD [41, 42]. Effective, accessible treatment options are desperately needed. Intervention trials are needed to identify effective treatment for those with stimulant and opioid co-use, as there are limited evidence-based, effective treatment options that address both. An exception is the use of contingency management, for which there is strong evidence supporting its use in outpatient programs for treating both opioid and stimulant use disorders [43–45], particularly in the context of a supplemental community reinforcement approach [46]. The efficacy of cognitive behavioral therapy for stimulant use disorders remains contested [46, 47], requiring more research, particularly with respect to polydrug use.

The fourth wave of the current overdose epidemic, characterized by combined use of opioids and stimulants, shows no sign of abating in the U.S. Our findings underscore a need to increase rural PWUD’s access to harm reduction tools including fentanyl test strips and overdose reversal medication, and for effective substance use treatment including MOUD. Rural PWUD are attempting to address this themselves, utilizing unregulated stimulants in the absence of adequate public health measures. Transcending the barriers to harm reduction and substance use treatment requires multi-faceted interventions. There is an urgent need for interventions tailored to people who use multiple substances, such as overdose education specific to the context of concurrent opioid and stimulant use, and fentanyl test strip and naloxone distribution targeted to persons who use stimulants. Finally, PWUD may be turning to methamphetamine to reverse overdose out of lack of awareness, lack of confidence, or confusion surrounding Good Samaritan Laws [48, 49]. Changing these misunderstadings requires broader educational outreach to rural PWUD, who may fear prosecution from calling emergency services [50, 51]. Lastly, implementation strategies and outreach efforts are urgently needed to improve MOUD access and retention to those with co-occurring opioid and stimulant use disorder living in rural areas.

## Study limitations

While we asked about current injection drug use of any type, we lacked a formal inquiry of current *non-IDU* stimulant use, which limited our ability to fully characterize modalities of stimulant use in rural areas. We did not probe on exact times of use, so discussions of concurrent use are necessarily simplified. Our data collection took place during the early stages of the fourth wave of the opioid overdose epidemic. As a result, our data may have included less mentions of unintentional opioid (e.g., fentanyl) use among stimulant users. Finally, we are limited by the relative racial and ethnic homogeneity of our sample which may obscure important differences in the experiences of PWUD who identify as nonwhite living in the rural U.S.

## Conclusion

In a rural U.S. cohort of rural persons who use opioids, a large proportion reported simultaneous stimulant and opioid use, as well as use of one drug while under the influence of the other. Key reasons for use included the pursuit of a unique psychoactive effect and attempting to experience the effects of opioids after developing a tolerance. Practical goals included offsetting the effects of the other drug, including avoidance or reversal of opioid-related overdose, and management of opioid withdrawal. Participants also sought increased energy/productivity for daily functioning and employment. The use of stimulants for harm reduction goals highlights the need for better access to harm reduction and polydrug treatment resources for PWUD in rural settings. Future qualitative and ethnographic research should focus on polydrug use, given the rapidly changing drug supply, and novel combinations of opioid and stimulant use.

## Data Availability

We welcome collaboration and encourage mentorship and the use of the ROI data stripped of all protected health information (PHI) to enable early investigators to address meaningful questions with support to help ensure their success. Additional information can be obtained at the ROI website: ruralopioidinitiative.org or by contacting the ROI DCC at ruralopioidinitiative@uw.edu. Follow the Rural Opioid Initiative on Twitter @ruralopioids.

## Abbreviations

IDU: Injection drug use
MOUD: Medications for opioid use disorder
NIDA: National Institute on Drug Abuse
PWUD: Person(s) who use(s) drugs
UW-DCC: University of Washington Data Coordinating Center
